# Cuproptosis in Ischemic Stroke: Molecular Mechanisms and Therapeutic Potential

**DOI:** 10.3390/molecules31183351

**Published:** 2026-09-21

**Authors:** Xinyu Han, Wenjing Zhang, Huanhuan An, Jianzhao Zhang, Jingwei Tian, Yunjie Wang

**Affiliations:** 1 School of Pharmacy, Key Laboratory of Molecular Pharmacology and Drug Evaluation (Yantai University), Ministry of Education, Collaborative Innovation Center of Advanced Drug Delivery System and Biotech Drugs in Universities of Shandong, Yantai University, Yantai 264005, China; 2 State Key Laboratory of Advanced Drug Delivery and Release Systems, Shandong Luye Pharmaceutical Co., Ltd., Yantai 264003, China

**Keywords:** ischemic stroke, cuproptosis, pharmacological, intervention

## Abstract

Stroke is a major neurological disease caused by cerebral vascular obstruction or rupture and represents a leading cause of death and long-term disability worldwide. Ischemic stroke (IS) accounts for the majority of stroke cases, while effective therapeutic strategies remain limited. Therefore, a better understanding of the underlying pathological mechanisms and the identification of new therapeutic targets are urgently needed. Cerebral ischemia triggers various regulated forms of cell death, including apoptosis, ferroptosis, and cuproptosis, which was discovered in recent years. Emerging evidence suggests that copper imbalance and copper-dependent cell death may play important roles in the development and progression of IS. This review summarizes the molecular mechanisms of cuproptosis and its potential involvement in cerebral ischemia, and discusses the therapeutic potential of cuproptosis-targeting agents for improving neurological outcomes after stroke.

## 1. Introduction

Stroke is a group of acute cerebrovascular disorders caused by the sudden rupture or blockage of blood vessels in the brain, leading to impaired cerebral blood flow and neurological deficits. It is a leading cause of death and long-term disability among adults worldwide [1]. Based on its pathological features, stroke is generally classified into ischemic stroke (IS) and hemorrhagic stroke [2]. According to the World Health Organization, millions of people die from stroke each year, while many survivors experience long-term disability [3]. As a result, stroke places a substantial economic and caregiving burden on patients, families, and healthcare systems. Its clinical symptoms are diverse and often develop suddenly, with rapid progression in some patients [4]. Current clinical management of IS primarily relies on intravenous thrombolysis and endovascular thrombectomy, whereas effective neuroprotective strategies remain limited. These approaches are intended to restore cerebral blood flow and preserve the ischemic penumbra [5]. However, their clinical use is limited by narrow treatment windows, the risk of ischemia–reperfusion injury [6], and the poor clinical translation of many neuroprotective agents. These limitations highlight the need to identify new therapeutic targets through a better understanding of IS pathophysiology [6].

Cellular demise is a pivotal event in the progression of cerebral ischemic injury. Beyond classical apoptosis, multiple regulated cell death pathways, such as necroptosis, pyroptosis and ferroptosis, have been implicated in neuronal loss following IS [7]. Recently, cuproptosis has been identified as a novel, copper-dependent form of regulated cell death, expanding our insights into the role of metal ion homeostasis in cellular fate [8]. Cuproptosis is a copper-dependent form of regulated cell death that is distinct from other known forms of cell death, including apoptosis, ferroptosis, pyroptosis, and necroptosis. It is primarily characterized by the direct binding of excess copper to lipoylated proteins of the tricarboxylic acid cycle (TCA cycle), leading to lipoylated protein aggregation, loss of iron–sulfur cluster (Fe-S cluster) proteins, proteotoxic stress, and ultimately cell death. Copper is an essential enzyme cofactor critical to numerous physiological processes, yet its dysregulation is linked to disease. Epidemiological studies indicate that dietary copper intake or plasma copper levels correlate positively with the risk of IS [9,10]. Following ischemia, sympathetic activation may further elevate serum copper levels, potentially exacerbating tissue damage [10]. These observations indicate that copper metabolism represents a potential source of diagnostic biomarkers for cerebral ischemia. A direct pathological link between copper-dependent cell death and cerebral ischemia is gradually emerging. Energy depletion caused by ischemia and hypoxia impairs the function of copper transporters, leading to increased intracellular copper levels and triggering copper toxicity. This phenomenon has been observed at 24 h after reperfusion in experimental models [11]. Crucially, preclinical studies indicate that pharmacological inhibition of cuproptosis, such as copper chelation therapy, reduces neuronal loss and improves functional recovery following ischemic injury [8]. This opens up a promising therapeutic avenue distinct from single-pathway neuroprotection, as it targets common mechanisms interlinked with cellular energy metabolism.

Although the research on cuproptosis is still in infancy stage in IS, drug development targeting this pathway provides new perspectives and strategic directions for treating major diseases. Thus, this review summarizes current knowledge on the etiology, classification, treatment of IS, and elaborates on the mechanisms of cuproptosis-mediated neuronal injury in the pathophysiology of IS and its therapeutic potential, while also reviewing candidate compounds with cuproptosis-inhibitory effects. The aim is to provide a reference for developing future therapeutic strategies.

## 2. Clinical Landscape and Pathophysiological Insights of Ischemic Stroke

### 2.1. Epidemiology of Stroke

Stroke remains a major public health concern in China, where more than two million new cases occur each year. It is also among the leading causes of disability-adjusted life years (DALYs) loss across all diseases [12]. According to the World Stroke Organization Global Stroke Fact Sheet 2025, approximately 7.8 million new IS cases and 3.6 million IS-related deaths were reported worldwide in 2021 [13]. When all stroke subtypes were considered, approximately 11.9 million incident strokes, 93.8 million prevalent cases, 7.3 million deaths, and 160.5 million DALYs were estimated worldwide in 2021. In the same year, stroke ranked as the third leading cause of death and the fourth leading cause of DALYs globally [14]. However, the burden of stroke is unevenly distributed across regions. Nearly 90 percent of stroke-related deaths and DALYs lost occur in low-income countries, highlighting major inequalities in the distribution of healthcare resources worldwide [13]. Stroke is not confined to older populations [13]. Around 15 percent of new stroke cases occur in people aged 15–49 years, while individuals younger than 70 years account for 53 percent of all new cases. These findings suggest that the onset of first-ever stroke is increasingly being observed in younger populations [13,14].

### 2.2. Etiology and Pathogenesis of Ischemic Stroke

**Figure 1:**
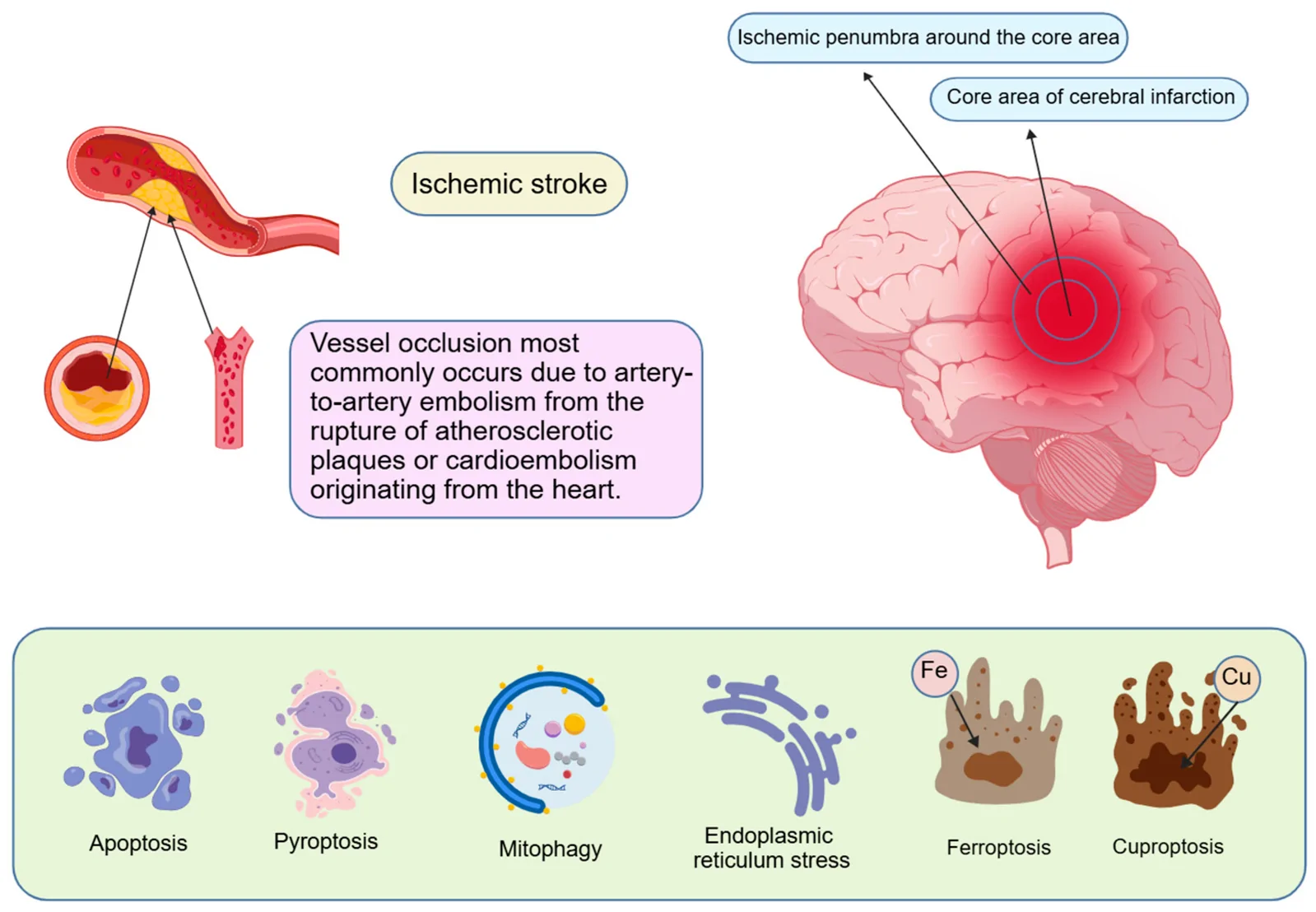
Mechanisms of vascular occlusion and regulated cell death mechanisms in ischemic stroke. In acute IS, the affected brain tissue is divided into the infarct core and the ischemic penumbra, with the infarct core located at the center of the ischemic lesion and the surrounding ischemic penumbra being a dynamically changing hypoperfused region. Its pathological damage mainly arises from vascular occlusion, thromboembolism, or cerebral hypoperfusion, leading to interrupted cerebral blood flow and a sharp decline in oxygen/glucose supply, while vascular rupture may induce hemorrhagic stroke. Cell death can occur through various pathways, including apoptosis, necroptosis, mitophagy, pyroptosis, endoplasmic reticulum (ER) stress, oxidative stress, ferroptosis, and disulfide-driven necrosis.

IS has a multifactorial etiology and is commonly classified according to the trial of org 10,172 in acute stroke treatment system into large-artery atherosclerosis, cardioembolism, small-vessel occlusion, stroke of other determined etiology, and stroke of undetermined etiology [15]. The core pathology begins with vascular obstruction, thromboembolism, or cerebral hypoperfusion, which interrupts cerebral blood flow and deprives brain tissue of oxygen and glucose as shown in Figure 1. Arterial occlusion most commonly results from atherosclerotic plaque rupture or cardioembolic events such as atrial fibrillation [16], while lacunar infarction due to small vessel disease represents another major subtype [17].

Following vascular occlusion, the affected brain tissue is conceptually divided into the infarct core and the ischemic penumbra. The infarct core experiences severe reductions in blood flow, resulting in rapid adenosine triphosphate (ATP) depletion and irreversible neuronal death, primarily through necrotic mechanisms. Surrounding this core area is the ischemic penumbra, a zone where blood flow is significantly reduced yet retains potential for salvage. Within this region, neuronal function is impaired and cellular survival is threatened, but structural integrity remains intact. The penumbra is the primary target for acute revascularization therapies, as timely reperfusion may rescue this tissue and improve functional outcomes. Conversely, without intervention, the penumbra progressively transforms into infarcted tissue, expanding the final lesion volume [16,18].

The damage caused by cerebral ischemia occurs in a segmental manner. Within minutes to hours, reduced cerebral blood flow triggers energy depletion, glutamate toxicity due to excessive release, and the collapse of ion gradients. This then progresses into the subacute phase (lasting from hours to days), characterized by massive production of reactive oxygen species (ROS), upregulation of adhesion molecule expression, activation of microglia, and intense inflammatory responses—reactions that are often exacerbated during reperfusion. Overall, these processes lead to various forms of regulated cell death (such as apoptosis and necroptosis), disruption of the blood–brain barrier (BBB), cerebral edema, and potential hemorrhagic transformation [19].

### 2.3. Clinical Manifestations and Treatment of Ischemic Stroke

The clinical presentation of IS primarily manifests as sudden onset of localized neurological deficits, with symptoms corresponding to the location and size of the infarct. Key symptoms include facial asymmetry, unilateral limb weakness, speech impairment (aphasia or dysarthria), and impaired balance or coordination, often accompanied by severe headache and dizziness. IS carries a substantial mortality risk. Survivors often face permanent disabilities including hemiplegia, sensory loss, aphasia, dysphagia, cognitive impairment, and emotional disorders. These conditions require extended rehabilitation and lead to a marked reduction in quality of life [19]. A transient ischemic attack produces similar clinical manifestations but resolves within a short period, it represents a key precursor to subsequent major ischemic events.

The management of IS is stage-dependent. Acute therapy focuses on restoring blood flow through two core strategies: intravenous thrombolysis and mechanical thrombectomy. These interventions aim to rescue the ischemic penumbra by reopening blocked vessels [19,20,21]. For secondary prevention, clinicians prescribe antiplatelet and cerebral metabolism-improving agents. Intravenous thrombolysis offers prognostic benefits, yet its application is restricted by a narrow therapeutic window, hemorrhage risk, and limited success in resolving large vessel blockages. As a result, nearly 50% of qualifying patients do not experience functional improvement, largely because of treatment timing issues, contraindications, and inadequate recanalization [18,22]. Therefore, there is an urgent need to develop more effective and faster-acting therapeutic strategies for IS.

## 3. Cuproptosis Mechanisms and Their Role in Ischemic Stroke

### 3.1. Regulation of Copper Metabolism

Copper is an essential trace element that participates in numerous physiological and biochemical reactions. In adults, total body copper averages approximately 100–200 mg, predominantly existing in the form of copper-binding proteins [23,24]. To prevent toxicity, cells maintain intracellular copper at very low concentrations through evolutionarily conserved homeostatic mechanisms. These dynamic regulatory processes actively counteract the concentration gradient to prevent the harmful accumulation of free copper, thereby preserving cellular copper homeostasis [25]. Both copper deficiency and excessive accumulation may lead to severe pathological consequences [26]. Copper deficiency can lead to severe anemia and may adversely affect systemic health [27]. Conversely, copper overload induces cell death and contributes to the onset and progression of diabetes, neurological disorders, and cardiovascular diseases.

The key to maintaining the aforementioned copper homeostasis lies in the precise uptake, distribution, and utilization of copper ions by cells, all of which depend primarily on the chemical forms of copper in the body and the mechanisms of its transmembrane transport. Copper exists in the body in two valence states, Cu^+^ and Cu^2+^. Cu^2+^ is the predominant form in the extracellular environment [28]. Before entering the cell, metal reductases located on the plasma membrane reduce Cu^2+^ to Cu^+^. If there is an excess of free Cu^+^ within the cell, fenton reaction will catalyze the production of highly reactive hydroxyl radicals, which cause oxidative damage to proteins, nucleic acids, and lipids, and interfere with the synthesis of Fe-S clusters. Intracellular copper levels are primarily regulated by copper transporter 1 (CTR1), a high-affinity copper uptake protein in the brain [29,30]. Upon entry into the cytoplasm, copper binds to specific proteins such as copper chaperone for superoxide dismutase (CCS) or superoxide dismutase 1 (SOD1) for delivery to distinct cellular compartments including mitochondria and the Golgi apparatus. SOD1 is an antioxidant protein that resides primarily in the cytoplasm. CCS regulates the distribution of SOD1 between the mitochondrial intermembrane space and the cytoplasm in an oxygen-dependent manner [31,32]. This regulatory mechanism maintains ROS homeostasis and alleviates ROS generated by the electron transport chain, thereby preventing oxidative damage caused by copper overload [31,32].

**Figure 2:**
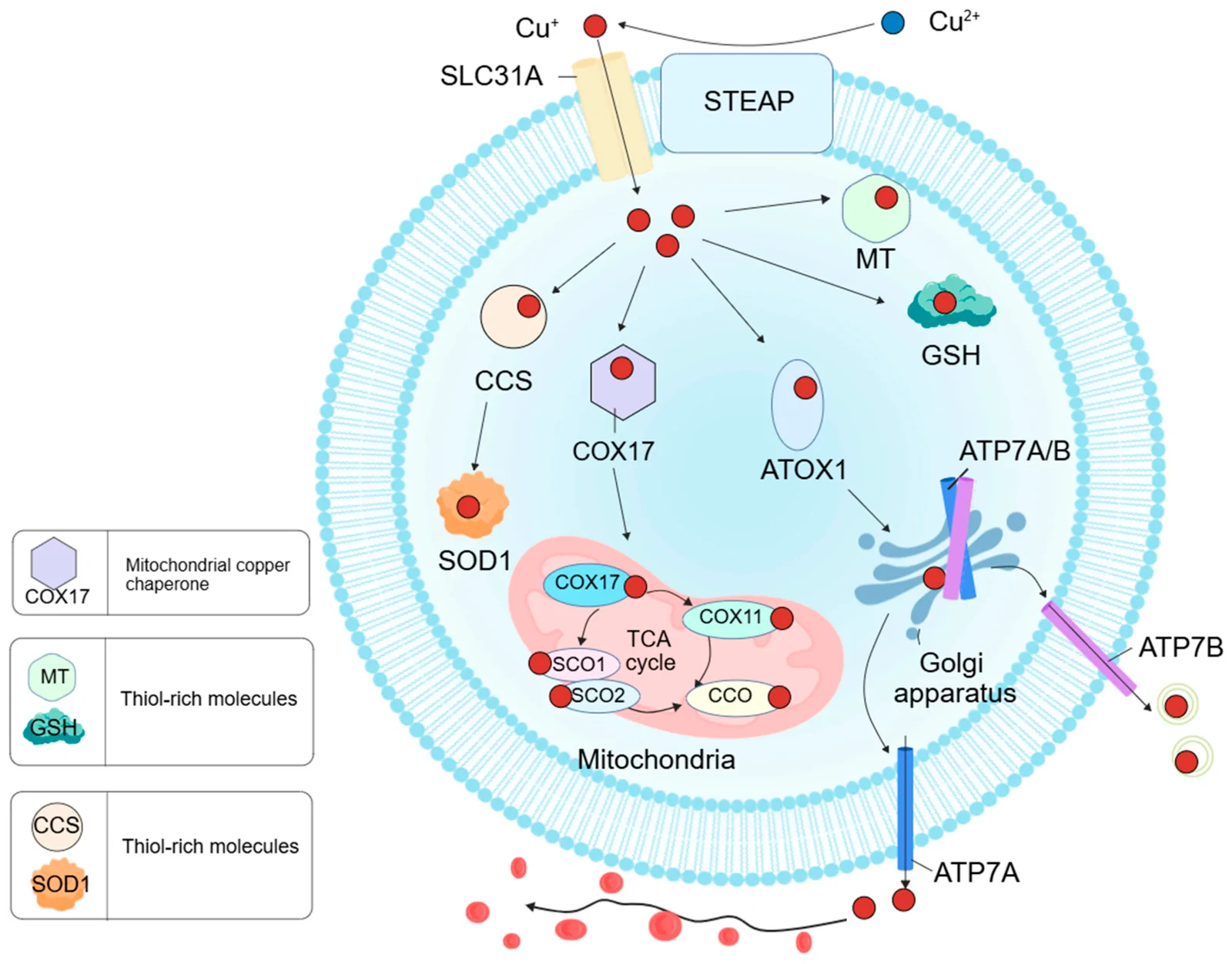
Intracellular copper metabolism pathway. Copper mainly exists as Cu^+^ and Cu^2+^ in the body. At the plasma membrane, Cu^2+^ is reduced to Cu^+^ by metal reductases such as the STEAP family. Cu^+^ then enters the cell through the high-affinity copper transporter SLC31A1, also known as CTR1. Because unbound copper may cause cellular damage, most intracellular Cu^+^ is rapidly taken up by copper-binding proteins. CCS delivers copper to SOD1 in the cytoplasm, supporting its antioxidant activity. COX17 carries copper into mitochondria and transfers it through proteins such as COX11, SCO1, and SCO2 for the assembly of cytochrome c oxidase (CCO), an important enzyme in the mitochondrial electron transport chain. ATOX1 transports copper to ATP7A or ATP7B in the Golgi apparatus, where copper is loaded into secreted copper-containing proteins. In enterocytes, ATP7A promotes copper export into the circulation and supports its transport to the liver, which is a major site of copper storage and regulation. In hepatocytes, ATP7B helps remove excess copper through vesicular transport and biliary excretion. Copper incorporated into specific proteins is then delivered to the cytoplasm, mitochondria, secretory pathway, or extracellular space, where it supports processes such as antioxidant defense, mitochondrial respiration, and collagen cross-linking. Excess intracellular Cu^+^ may also be bound and stored by GSH and MTs, thereby limiting copper-induced toxicity. STEAP: Six-Transmembrane Epithelial Antigen of the Prostate; SLC31A1: Solute carrier family 31 member 1; CTR1: Copper transporter 1; CCS: Copper chaperone for superoxide dismutase; SOD1: Superoxide dismutase 1; COX17: Cytochrome c oxidase assembly protein 17; COX11: Cytochrome c Oxidase Copper Chaperone 11; SCO1: Synthesis of Cytochrome c Oxidase 1; SCO2: Synthesis of Cytochrome c Oxidase 2; CCO: cytochrome c oxidase; ATOX1: Antioxidant 1 Copper Chaperone; ATP7A: ATPase Copper Transporting Alpha; ATP7B: ATPase copper transporting beta; GSH: Glutathione; MTs: Metallothioneins.

In humans, the copper absorption rate is inversely proportional to intake, generally ranging between 40% and 60%. Most unabsorbed copper is excreted via feces and urine, while a small fraction undergoes reabsorption and reuse within the digestive tract [33,34]. As shown in Figure 2, Antioxidant 1 copper chaperone (ATOX1) functions as an intracellular copper transporter, delivering Cu^+^ to copper-transporting adenosine triphosphatases (ATPases). Inside the cell, copper binds to cuproproteins via the copper-transporting ATPase copper transporting alpha (ATP7A) and ATPase copper transporting beta (ATP7B), located in the trans-Golgi network (TGN), and is subsequently exported out of the cell in vesicular form [35]. Temporary copper storage relies primarily on thiol-rich molecules such as metallothionein (MT) and glutathione (GSH). Both MT and GSH form reversible bonds with free copper ions, establishing a molecular buffering system that prevents free diffusion and toxic injury within the cell [36,37]. Under copper-overload conditions, cells typically upregulate ATP7A and ATP7B expression to enhance transmembrane copper transport while promoting MT synthesis. This dual mechanism achieves effective copper chelation and excretion, thereby maintaining copper homeostasis [33].

Upon delivery to the TGN, copper ions are sequestered by ATP7A/B, which catalyzes the maturation of cuproenzymes, including ceruloplasmin, lysyl oxidase, and tyrosinase. ATP7A and ATP7B exhibit distinct tissue-specific expression patterns. While ATP7A is broadly expressed across most organs excluding the liver, ATP7B is primarily restricted to hepatic tissue [38]. In the presence of excess copper, cells typically enhance copper transport across the membrane by promoting the translocation of ATP7A and ATP7B from the TGN to the plasma membrane or vesicles. At the same time, they upregulate the synthesis of metallothioneins to facilitate efficient copper chelation and efflux, thereby maintaining copper homeostasis [39]. In addition, cytochrome c oxidase assembly protein 17 (COX17), a mitochondrial copper chaperone, delivers copper ions to the inner mitochondrial membrane and participates in the assembly of cytochrome c oxidase (COX) subunits, thereby ensuring the proper function of the mitochondrial electron transport chain [40]. In summary, various copper-binding proteins work together with transporters to maintain intracellular copper homeostasis.

Under physiological conditions, copper-related proteins maintain dynamic copper ion homeostasis, which is tightly regulated by proteins such as CTR1, ATP7A/B, ATOX1, and CCS. This ensures copper functions as a cofactor in essential metabolic processes including oxidative phosphorylation and antioxidant defense, while preventing toxic accumulation. In various pathological models of copper ion accumulation, copper homeostasis is disrupted, and the expression or function of copper-related proteins undergoes adaptive or pathological changes: copper transporters such as Solute carrier family 31 member 1 (SLC31A1) and ATP7A/ATP7B are upregulated [41], and the cuproptosis upstream regulator ferredoxin 1 (FDX1) and downstream effector lipoyl synthase (LIAS) show a marked upregulation trend [42].

### 3.2. Mechanism of Cuproptosis

Tsvetkov et al. [8] first discovered cuproptosis in 2022. They found that cuproptosis was distinct from known cell death mechanisms such as apoptosis, necroptosis, pyroptosis, and ferroptosis, representing a novel form of cell death. The researchers [8] further demonstrated that reducing the copper-binding capacity of these compounds markedly diminished their cell-killing activity, indicating that copper binding is essential for copper ionophore-induced cell death. Subsequently, they evaluated the killing potential of the potent copper ionophore elesclomol, which showed that cells cultured in copper-deficient medium exhibited resistance to the drug, whereas sensitivity was completely restored by the addition of copper and supplementation with other metals did not produce this effect. It was concluded that elesclomol forms a lipophilic complex with Cu^2+^ in the extracellular space, enabling it to cross both the plasma membrane and the mitochondrial membrane. In the reductive environment of the mitochondria, Cu^2+^ is reduced to Cu^+^ and released, leading to abnormal accumulation of active copper in the mitochondrial matrix. This accumulation serves as the initiating event that triggers cuproptosis.

Moreover, cells exhibiting a high reliance on mitochondrial respiration display heightened sensitivity to cuproptosis, underscoring a critical link between this cell death modality and the TCA cycle. Building on this, the researchers further proposed the concept that “mitochondrial respiration regulates cuproptosis”: Copper ions directly bind to lipoylated proteins in the TCA cycle, causing them to aggregate abnormally, which in turn leads to the loss of Fe-S cluster and proteotoxic stress, ultimately triggering cell death [8]. Experiments have shown that, following inhibition of mitochondrial respiration, copper-induced cell death is significantly suppressed, while other death pathways, such as ferroptosis, remain unaffected, indicating that mitochondrial respiration is a necessary condition for copper-induced cell death [43]. Lipoylation is a highly conserved post-translational modification of lysine residues on key enzymes of the TCA cycle, regulated by proteins such as FDX1 and LIAS [43,44]. Excessive binding of copper ions to these lipoylated proteins not only leads to their aggregation but also disrupts mitochondrial metabolic homeostasis, including the inhibition of Fe-S cluster synthesis and the disruption of electron transport chain function, thereby triggering an energy crisis and oxidative stress [8]. Although the accumulation of ROS may accompany the process of copper-mediated cell death and exacerbate mitochondrial damage, current evidence suggests that the core mechanism of copper-mediated cell death is proteotoxic stress rather than an ROS-driven apoptotic cascade [8,45]. Proteotoxic stress serves as a pivotal link in the execution of cuproptosis. It refers to a pathological state in which the excessive accumulation of misfolded or aberrantly aggregated proteins overwhelms the capacity of cellular quality control systems, ultimately leading to cellular dysfunction. Studies have demonstrated that in both Atp7B heterozygous mice and wild-type control mice, a marked loss of lipoylated and Fe-S cluster proteins, accompanied by increased abundance of Heat shock protein 70 (HSP70), can be clearly observed [8]. These findings establish proteotoxic stress as a central, intermediate executor in the lethal cascade triggered by copper ions.

**Figure 3:**
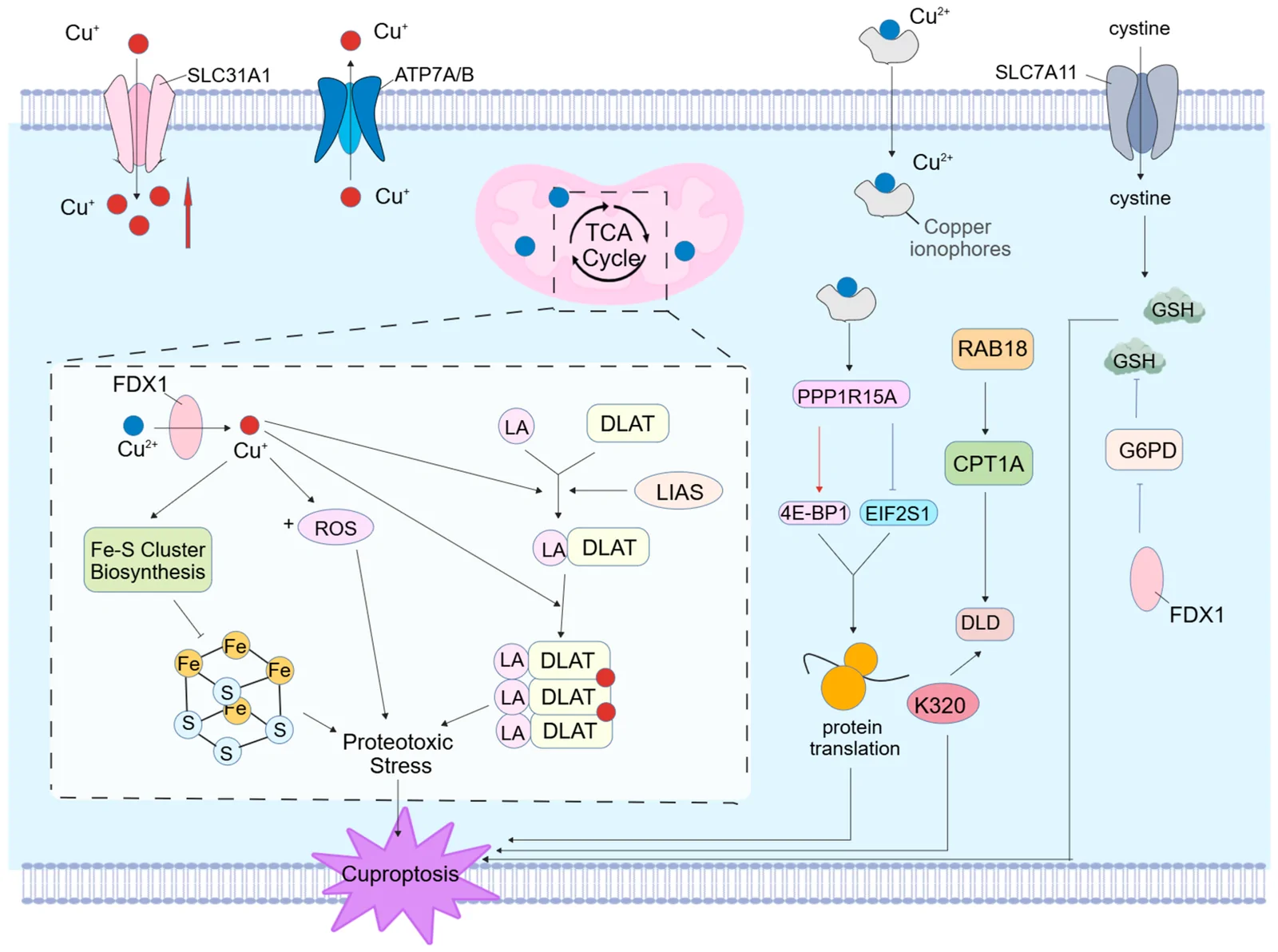
Molecular mechanisms underlying cuproptosis. Under normal conditions, Cu^+^ enters cells through SLC31A1, whereas ATP7A/B helps export excess copper and maintain intracellular copper balance. Copper ionophores, such as elesclomol, may also carry Cu^2+^ into cells and increase mitochondrial copper levels. In mitochondria, FDX1 converts Cu^2+^ to Cu^+^. The resulting Cu^+^ binds to lipoylated DLAT, promotes DLAT aggregation, and disrupts iron–sulfur cluster proteins. Together with increased ROS production, these changes cause mitochondrial proteotoxic stress and finally lead to cuproptosis. Several other pathways can further strengthen this process. FDX1 promotes the ubiquitination and degradation of G6PD, which lowers GSH levels and weakens cellular antioxidant defense. The SLC7A11-mediated uptake of cystine also affects GSH synthesis and therefore influences the sensitivity of cells to copper-induced death. In addition, RAB18 suppresses lipophagy and increases CPT1A expression, leading to K320 succinylation of DLD and promoting cuproptosis. PPP1R15A further enhances this response by reducing eIF2α phosphorylation and increasing 4EBP1 phosphorylation, thereby accelerating protein synthesis and aggravating proteotoxic stress. SLC31A1: Solute carrier family 31 member 1; ATP7A/B: ATPase Copper Transporting Alpha/Beta; FDX1: Ferredoxin 1; DLAT: Dihydrolipoamide S-acetyltransferase; ROS: Reactive Oxygen Species; G6PD: Glucose-6-Phosphate Dehydrogenase; GSH: Glutathione; CPT1A: Carnitine palmitoyltransferase 1A; DLD: dihydrolipoamide dehydrogenase; PPP1R15A: Protein phosphatase 1 regulatory subunit 15A; 4EBP1: 4E binding protein 1.

As shown in Figure 3, FDX1 plays a key role in mitochondrial metabolism by promoting lipoylation through direct binding to and activation of the acyl-synthetase LIAS [8]. It acts as an upstream regulator of cuproptosis by directly binding to LIAS and promoting protein lipoylation [46]. FDX1 facilitates mitochondrial copper reduction and promotes LIAS-dependent lipoylation of TCA cycle proteins. Loss of FDX1 decreases protein lipoylation and subsequently reduces the susceptibility to cuproptosis [46,47]. Meanwhile, Cu^2+^ can form stable coordination complexes with the lipoylated TCA cycle-related enzymes dihydrolipoamide S-acetyltransferase (DLAT), dihydrolipoamide S-succinyltransferase (DLST), and the branched-chain α-ketoacid dihydrolipoamide branched-chain transacylase E2, inducing aggregation of lipoylated proteins and disrupting mitochondrial proteostasis. In addition, cuproptosis is accompanied by the loss of Fe-S cluster proteins, which further contributes to mitochondrial dysfunction. This subsequently inhibits the function of key electron transport-related proteins such as LIAS, directly interferes with the activity of mitochondrial respiratory chain complexes, reduces ATP synthesis efficiency, and induces mitochondrial stress. Subsequent mitochondrial structural damage then drives cells into a copper-dependent death program.

In addition, excess copper ions compete with Fe-S cluster proteins for coordination, leading to Fe-S cluster dissociation. This not only directly impairs the function of key electron transport–related proteins, such as lipoyl synthase (LIAS), but also disrupts the activity of mitochondrial respiratory chain complexes, further reducing ATP synthesis efficiency and thereby triggering a pronounced mitochondrial stress response [46]. Ultimately, such mitochondrial structural damage under these combined insults forces cells to undergo a copper-dependent cell death program.

Beyond canonical mitochondrial respiratory chain proteins, Ras-related protein (RAB18) also induces cuproptosis [48]. RAB18 is a small GTPase localized at the endoplasmic reticulum/lipid droplet interface that mediates lipid droplet homeostasis in hepatic stellate cells. Carnitine palmitoyltransferase 1A (CPT1A) is recruited into this microenvironment and promotes the succinylation of dihydrolipoamide dehydrogenase (DLD) at site K320. K320 is also a competitive site for succinylation and ubiquitination: succinylation at this lysine impedes K48-linked ubiquitination, thereby inhibiting DLD degradation via the proteasome and extending its half-life, leading to stable accumulation of DLD within the cell. Concurrently, GSH depletion weakens copper chelation buffering, resulting in the upregulation of the copper transporter SLC31A1 and downregulation of ATP7A and ATP7B. FDX1 mediates the reduction in Cu2+ to Cu+, enabling the enrichment of highly reactive Cu+ in the mitochondrial matrix abundant with lipoylated proteins [48].

Elesclomol-Cu induces mitochondrial stress and aggregation of lipoylated proteins upon entering cells, thereby upregulating the stress-associated regulatory subunit Protein phosphatase 1 regulatory subunit 15A (PPP1R15A) in an FDX1/DLAT-dependent manner [49]. PPP1R15A itself lacks catalytic activity. Instead, it binds protein phosphatase 1 (PP1) to alter its substrate selectivity, forming the PPP1R15A-PP1 regulatory complex. On one hand, this complex dephosphorylates eukaryotic translation initiation factor 2 subunit alpha at serine 51, relieving global translational repression under integrated stress. On the other hand, it maintains or promotes phosphorylation of eukaryotic translation initiation factor 4E binding protein 1 (4EBP1) at threonine 70, enabling release of eukaryotic translation initiation factor 4E and enhancing cap-dependent translation initiation and influx of nascent proteins [49]. At this stage, mitochondria sustain irreversible damage and perturbation of the respiratory chain. The renewed acceleration of translation significantly increases the folding burden of nascent polypeptides, which, compounded with preexisting proteotoxic stress marked by aggregation of lipoylated proteins and upregulation of HSP70, amplifies proteotoxic stress and ultimately drives cells into the copper-dependent cell death program [49]. In addition, FDX1 interacts with glucose-6-phosphate dehydrogenase (G6PD) and promotes its ubiquitination, thereby reducing the protein stability and enzymatic activity of G6PD. This decreases reduced nicotinamide adenine dinucleotide phosphate production and limits GSH regeneration. The resulting decline in GSH weakens cellular redox defense and the ability to chelate Cu+, thereby promoting cuproptosis [50].

### 3.3. The Role of Cuproptosis in the Pathological Process of Stroke

Imbalances in essential metals such as iron, zinc, and copper are linked to numerous neurological diseases [51]. Physiologically, copper maintains brain health. However, following stroke, disruption of copper homeostasis may contribute to neuronal injury by promoting copper-dependent cell death pathways, including cuproptosis [8,52]. Studies indicate that serum copper levels are significantly elevated in patients with cerebral ischemia [10]. Due to the uneven distribution of copper in the brain, the locus coeruleus region of brainstem neurons exhibits the highest concentration [53]. This region serves as the primary source of norepinephrine in the central nervous system. One possible explanation for the elevated serum copper in cerebral ischemia is that during acute IS, the body enters a stress state, sympathetic nerves are activated, and catecholamine secretion increases. Because dopamine β-hydroxylase, a copper-dependent enzyme involved in catecholamine synthesis, requires copper as a cofactor, sympathetic activation may influence systemic copper metabolism [10]. Cerebral ischemia leads to hypoxia, which decreases mitochondrial ATP production, limits copper transport, and results in copper accumulation. During cerebral ischemia–reperfusion, the balance between intracellular copper uptake and efflux may become disrupted [28,54]. Therefore, inhibiting neuronal cuproptosis may alleviate copper-induced mitochondrial damage and reduce neuronal loss during cerebral ischemia–reperfusion.

Clinical observations and basic research together reveal a complex association between copper metabolism and the risk of IS. On one hand, insufficient dietary copper intake may increase the risk of stroke, indicating the importance of maintaining adequate copper intake [52]. On the other hand, in the acute phase of IS, copper ion levels in serum and cerebrospinal fluid are significantly elevated and show a positive correlation with the degree of neurological injury [10]. Genome-wide association analyses have further revealed that the expression levels of cuproptosis-related genes such as NLR family pyrin domain containing 3, NFE2 like bZIP transcription factor 2, ATP7A, lipoyltransferase 1, glutaminase, and metal regulatory transcription factor 1 in IS patients are significantly correlated with the infiltration levels of immune cells including monocytes and neutrophils, suggesting a close association between dysregulation of copper homeostasis and the modulation of the post-IS immune microenvironment [55]. Collectively, these lines of evidence indicate that precise regulation of copper homeostasis is central to therapeutic intervention.

Cuproptosis plays a pivotal role in the pathophysiology of IS. Cerebral ischemia interrupts blood flow and forces a metabolic shift from oxidative phosphorylation to glycolysis, leading to ATP depletion, calcium overload, excitotoxicity, and oxidative stress [18]. This metabolic crisis promotes abnormal copper accumulation within mitochondria. Excess copper binds to lipoylated proteins in the tricarboxylic acid cycle, triggering toxic protein aggregation and the loss of iron–sulfur cluster proteins. This cascade induces mitochondrial proteotoxic stress, culminating in cuproptosis and amplifying neuronal damage [8,28]. Different cell types exhibit distinct sensitivities to this process. Neurons are highly vulnerable to copper toxicity, as copper directly induces cuproptosis by disrupting the cAMP response element-binding protein (CREB) signaling pathway [56]. When cuproptosis occurs in astrocytes, these cells release substantial amounts of copper, exerting secondary toxicity on neighboring neurons [57]. Cuproptosis and endothelial injury have been associated with blood–brain barrier disruption after cerebral ischemia, which may further disturb cerebral copper homeostasis and aggravate ischemic brain injury [57,58]. Beyond direct neuronal loss, cuproptosis also disrupts the microenvironment necessary for neural regeneration, severely hindering long-term functional recovery after stroke [59,60].

More importantly, emerging evidence suggests that inhibition of cuproptosis-related processes can alleviate cerebral ischemia/reperfusion (I/R) injury. Ding et al. [61] established a mouse Middle Cerebral Artery Occlusion/Reperfusion (MCAO/R) model and found that cerebral I/R was accompanied by increased Cu^2+^ levels and elevated expression of the cuproptosis-related proteins HSP70, LIAS, and DLST. Methyltransferase-like 14 (METTL14) knockdown or treatment with the copper chelator tetrathiomolybdate (TTM) improved neurological deficits and reduced cerebral infarct volume. Importantly, administration of the cuproptosis inducer elesclomol to MCAO/R mice with METTL14 knockdown restored Cu^2+^ accumulation and the expression of cuproptosis-related proteins, while aggravating neurological deficits and cerebral infarction, thereby partially reversing the protective effects of METTL14 knockdown.

Recent studies have suggested an association between disturbances in copper homeostasis and ischemic stroke; however, direct evidence that cuproptosis triggers stroke onset is still lacking [38]. Given that cuproptosis is a recently identified form of regulated cell death, we speculate that it may contribute to the onset or progression of some cases of ischemic stroke. Nevertheless, there is currently insufficient evidence to establish cuproptosis as an independent cause of cryptogenic stroke or stroke of undetermined etiology. Based on current evidence, cuproptosis is more likely to act as a downstream mechanism contributing to neuronal injury after cerebral ischemia and reperfusion. Therefore, “cuproptosis-mediated ischemic stroke” is currently better understood as ischemic brain injury involving cuproptosis, while whether a distinct stroke subtype directly driven by cuproptosis exists requires further investigation.

Cuproptosis serves as a central pathological link between acute ischemic injury and impaired long-term repair. The core strategy lies in precisely inhibiting cuproptosis by intervening in copper ion transport, applying specific copper chelators, or regulating downstream key proteins. Blocking this pathway may reduce neuronal death during the acute phase while simultaneously creating favorable conditions for neural regeneration, thereby offering a novel, comprehensive intervention strategy to improve the prognosis of IS. Future research should focus on the specific regulatory mechanisms of cuproptosis in IS and promote the clinical translation of related inhibitors.

## 4. Translational Therapeutic Strategies Targeting Cuproptosis Across Disease Contexts

### 4.1. Small Molecule Drugs Inhibiting Cuproptosis for the Treatment of Ischemic Stroke

Targeted copper delivery strategies have been demonstrated to alleviate neuronal injury induced by IS [62]. During cerebral ischemia–reperfusion, the homeostatic balance between intracellular copper uptake and efflux is disrupted due to impaired mitochondrial respiratory chain function and limited ATP generation. Sustained copper accumulation may induce oxidative stress damage in cerebral ischemia–reperfusion injury. In the phase of cerebral ischemia–reperfusion, inhibiting neuronal cuproptosis may reduce intracellular copper levels and alleviate neuronal injury [28]. Copper chelators maintain intracellular copper homeostasis, which is particularly important in the treatment of certain diseases [38], such as glioma, Alzheimer’s disease, and Huntington’s disease.

However, the non-specificity and potential toxicity of copper chelators limit their clinical application. Excessive chelation may lead to essential copper deficiency, affecting the physiological functions of copper-dependent enzymes [63]. Studies have proven that certain copper chelators are not effective in treating stroke. Ambi et al. [64] evaluated the therapeutic effect of the copper-specific chelator tetrathiomolybdate (TTM) in a transgenic rat model (rTg-DI) of cerebral amyloid angiopathy (CAA), a disease characterized by amyloid-β (Aβ) protein deposition in cerebral blood vessels, which often leads to cerebral hemorrhage [65], stroke [66], and dementia [67]. The study found that TTM effectively removed copper bound to Aβ in vitro, but failed in vivo due to TTM retention and copper redistribution. Therefore, intraperitoneal administration could not effectively clear copper from vascular Aβ deposits. Instead, it exacerbated CAA pathology by promoting copper accumulation.

GSH, a tripeptide conjugate synthesized from glutamate, cysteine, and glycine, serves as a pivotal player in safeguarding cellular redox homeostasis, orchestrating detoxification pathways, and bolstering antioxidant defenses. Liu et al. [68] found that GSH directly chelates copper ions within mitochondria, reducing toxic free copper levels and thereby antagonizing Elesclomol-Cu induced cuproptosis in pancreatic ductal adenocarcinoma cells. The cuproptosis inducer unexpectedly activated a robust selfprotective pathway by stabilizing the transcription factor nuclear factor erythroid 2-related factor 2, upregulating the expression of the rate-limiting GSH synthesis enzymes glutamate–cysteine ligase modifier subunit and glutamate–cysteine ligase catalytic subunit, and promoting GSH synthesis. Newly synthesized GSH is then specifically transported into mitochondria via the mitochondrial transporter solute carrier family 25 member 39, where it directly chelates excess copper ions within the mitochondrial lumen, thereby relieving copper toxicity and resisting cuproptosis.

Notably, the inhibition of cuproptosis has also been demonstrated to be a mechanism by which cells acquire a survival advantage under certain pathological conditions. Wang et al. [69] discovered that in clear cell renal cell carcinoma, elevated expression of adrenomedullin (ADM) inhibited the expression of the key cuproptosis protein FDX1 by activating the p38 mitogen-activated protein kinase-forkhead box O3 signaling axis, thereby suppressing cuproptosis, which ultimately led to tumor cell resistance to sunitinib.

Dexmedetomidine (Dex), a highly selective and efficacious α_2_-adrenoreceptor agonist, has been widely incorporated into clinical regimens for the management of sedation and analgesia [70]. Focusing on cuproptosis, Guo et al. [11] elucidated the mechanism of Dex in cerebral ischemia–reperfusion injury. Employing a rat cerebral ischemia–reperfusion model, the investigators assessed the impact of Dex and the copper chelator D-penicillamine on infarct size, copper homeostasis, mitochondrial bioenergetics (respiration and membrane potential), glutathione reserves, and the expression profiles of core cuproptosis-associated proteins. The study demonstrated that Dex conferred cytoprotection by suppressing the canonical FDX1-mediated signaling cascade, revealing its pleiotropic neuroprotective effects against cerebral ischemia–reperfusion injury.

Edaravone dextrose (EDB), a neuroprotective agent, is extensively employed in the clinical management of cerebral infarction, primarily owing to its established anti-inflammatory and free radical scavenging capabilities [71,72]. Jin et al. [58] evaluated the effects of EDB on ferroptosis, cuproptosis, and BBB disruption after cerebral infarction in vivo and in vitro by constructing a mouse middle cerebral artery occlusion (MCAO) model and oxygen–glucose deprivation/reperfusion (OGD/R) models of neurons and brain microvascular endothelial cells. Data indicated that EDB treatment activated the solute carrier family 7 member 11 (SLC7A11)/Glutathione Peroxidase 4 axis—a critical protective pathway against ferroptosis—while concurrently suppressing the expression of cuproptosis-enhancing proteins, specifically SLC31A1 and FDX1. In addition, EDB improved neurological function, reduced brain water content and cerebral infarct volume, and alleviated BBB damage. By targeting key cuproptosis proteins and copper ion homeostasis, EDB demonstrated feasibility for treating stroke at the experimental level.

Cuproptosis inducers are currently the key drug category in research on cuproptosis, which are commonly used in tumors, inducing cuproptosis in cells also induces cuproptosis in cancer cells. 8-Hydroxyquinolines (8HQs) are a class of metal ion chelators that have been widely used in pharmaceutical applications since they were first prepared in the early 1870s. 8HQ binds to Cu^2+^ extracellularly to form a lipophilic complex. This complex passively diffuses across the cell membrane, transporting substantial amounts of copper ions into the cell and increasing intracellular copper accumulation and cytotoxicity [73]. Therefore, we hypothesize that blocking the formation of this carrier complex may prevent the generation of the toxic entity and subsequently inhibit the occurrence of cuproptosis. Zinc pyrithione (ZnPT) is a topical antimicrobial agent approved by the US Food and Drug Administration [74]. It functions as a copper ionophore, inducing intracellular copper influx and iron–sulfur protein inactivation, thereby inhibiting fungal growth [75,76]. Yang et al. [77] found that ZnPT transported extracellular copper ions into cells, significantly increased intracellular copper ion levels, disrupted copper homeostasis, and within mitochondria, the accumulated copper ions directly bound to the lipoylated protein DLAT in the TCA cycle, inducing its focal aggregation and oligomerization. These cascade reactions ultimately triggered proteotoxic stress and induced cuproptosis. Therefore, in developing inhibitors of ZnPT, one strategy is to design blockers of its transport function. Such agents competitively inhibit the binding of ZnPT to proteins essential for its transport on the cell membrane or mitochondrial membrane, thereby block its ionophore activity and prevent the delivery of copper ions to mitochondrial targets, thus suppress cuproptosis.

The small molecule drugs discussed above, together with their mechanisms of action and experimental models, are systematically summarized in Table 1.

### 4.2. Traditional Chinese Medicine and Natural Products Inhibiting Cuproptosis for Ischemic Stroke Treatment

It is well established that the inhibition of oxidative stress, apoptosis, and inflammatory responses [78,79,80], as well as promotion of neurogenesis [81] are some of the mechanisms underlying anti-brain ischemia medications. These therapeutic effects are attributable to the principal bioactive constituents derived from traditional Chinese medicine (TCM). Investigations into TCM monomers for IS have predominantly centered on the identification of their bioactive ingredients and effective fractions [82]. Natural products, with their multi-target and multi-dimensional regulatory properties, are particularly well-suited to the complexity of the cuproptosis pathway and have rapidly become a research hotspot targeting this pathway.

Corymbinol A is a natural active compound isolated from the fruit of *Corymbia citriodora*, belonging to the bis-β-triketone-sesquiterpene hybrid class. Li et al. [83] experimentally demonstrated that Corymbinol A, at a non-cytotoxic concentration of 20 μM, significantly inhibited elesclomol/CuCl_2_ induced cuproptosis with an inhibition rate of 31%, an effect comparable to the positive control TTM. Moreover, Corymbinol A protected mitochondrial function by maintaining the stability of Fe-S proteins in the TCA cycle, thereby increasing aconitase 2 (ACO2) expression levels. Importantly, the inhibitory effect of Corymbinol A on cuproptosis was highly specific, underscoring its important research value for targeting cuproptosis in IS treatment.

*Astragalus membranaceus* (Huangqi) is defined as the desiccated root derived from two botanical species: *Astragalus mongholicus Bunge* or *Astragalus membranaceus* (Fisch.) Bunge, has a history of application spanning over two thousand years [84]. Although direct literature investigating the relationship between Astragaloside IV (AS) and cuproptosis is limited, the multi-dimensional protective effects of AS on mitochondria suggest that it may indirectly or directly inhibit cuproptosis, thereby achieving multi-target therapeutic effects for IS. Wang et al. [85] found that AS treatment restored mitochondrial homeostasis and ER function. Furthermore, they systematically elucidated that ASmodulated the dual specificity phosphatase 1 (DUSP1)-prohibitin 2 (PHB2) axis, intervening in mitochondrial quality control (MQC) at multiple levels, thereby improving mitochondrial energy metabolism and oxidative stress, regulating mitochondrial dynamics, and enhancing mitophagy.

Calycosin-7-O-β-D-glucoside (CG) is a characteristic component of *Astragali Radix* (AR) and belongs to an isoflavone, and its content is relatively high among the flavonoids of AR [86]. Lou et al. [87] established the OGD/R model of SH-SY5Y cells and the middle cerebral artery occlusion/reperfusion (MCAO/R) model of Sprague-Dawley rats. The study found that by inhibiting the glycogen synthase kinase 3β (GSK-3β)/activating transcription factor 3 (ATF3)/SLC31A1 pathway, CG significantly boosted OGD/R cell survival, promoted neuronal cell growth, attenuated nerve damage in MCAO/R rats, and reversed the abnormal changes in cuproptosis proteins.

*Forsythia suspensa* is a traditional Chinese medicinal herb commonly used for clearing heat and detoxification. Its main active components include forsythin, forsythoside, and phillygenin (PHI). Recent studies have found that the active components in *Forsythia suspensa* exhibit neuroprotective potential for treating neurodegenerative and other neurological diseases. Their mechanisms of action involve multiple signaling pathways, demonstrating neuroprotective potential in the treatment of neurodegenerative and other neurological disorders [88]. Chen et al. [89] discovered that PHI could reduce cuproptosis in myocardial ischemia–reperfusion injury by regulating the degradation of CTR1. Myocardial and cerebral ischemia share similar mechanisms of reperfusion injury, particularly the common role of mitochondrial dysfunction in ischemia–reperfusion injury across various organs. Yan et al. [90] established a rat model of middle cerebral artery occlusion and found that PHI significantly reduced cerebral infarct size in the rats, and also significantly inhibited microglial activation and reduced apoptosis. These findings provided a theoretical basis for exploring the use of *Forsythia suspensa* in treating IS by inhibiting cuproptosis. In addition, PHI has significant antioxidant stress and metal ion chelation abilities. This drug may alleviate tissue damage by regulating metal ion homeostasis.

Artesunate (ART) is a semi-synthetic derivative of artemisinin extracted from *Artemisia annua* L. [91], possessing pharmacological effects such as antiviral, antitumor, antioxidant, and anti-inflammatory activities [92,93]. Chen et al. [94] investigated the neuroprotective effect of ART on cerebral I/R injury and its underlying mechanism, and found that ART significantly reduced infarct volume, suppressed myeloperoxidase activity, and decreased the expressions of Toll-like receptor-4, myeloid differentiation primary response 88, nuclear factor-kappa B (NF-κB), tumor necrosis factor-α, and interleukin-6 in the ischemic cortex area. Following the discovery of cuproptosis, studies have shown that ART can effectively inhibit this form of cell death. Cheng et al. [95] first discovered that ART directly bound to the metallothionein 2A (MT2A) protein in astrocytes, enhanced its copper-chelating capacity, and thereby regulated cuproptosis. This led to a reduction in intracellular Cu^2+^ levels, effectively restored copper transport, and downregulated core cuproptosis driver proteins. Specifically, the expression of the copper uptake protein CTR1 was restored or increased, while FDX1 expression was decreased.

Xinfeng Capsule (XFC) is a standardized TCM formulation pioneered by the First Affiliated Hospital of Anhui University of Traditional Chinese Medicine. The principal bioactive constituents encompass calycosin-7-O-β-D-glucoside, calycosin, formononetin, β-sitosterol, and stigmasterol, which collectively underlie its anti-inflammatory, antioxidant, and immunomodulatory properties [96]. Wang et al. [97] first demonstrated that XFC inhibited cuproptosis and inflammation in chondrocytes via the methyltransferase like 3/microRNA-221/222-3p/ATPase copper transporting alpha axis (METTL3/miR-221/222-3p/ATP7A axis), exerted protective effects against rheumatoid arthritis-associated joint injury, provided novel mechanistic insights into the therapeutic actions of XFC, and offered a promising strategy for the treatment of rheumatoid arthritis. This study reveals that the METTL3/miR-221/222-3p/ATP7A axis constitutes a druggable endogenous anti-cuproptosis pathway.

Trilobatin (TLB) is a naturally occurring antioxidant derived from the leaves of *Lithocarpus polystachyus Rehd*, which is generally recognized as safe and has been recently approved by the Food and Agriculture Organization as a food additive [98]. Studies found that TLB possessed strong antioxidant properties and exhibited a variety of pharmacological activities, including neuroprotective, anti-diabetes, anti-aging, and hepatoprotective effects [99,100]. Wei et al. [101] found that TLB inhibited cuproptosis via the FDX1-mediated copper-dependent cell death signaling pathway in both in vivo doxorubicin-induced cardiotoxicity (DIC) mouse models and in vitro Doxorubicin-induced H9c2 cell injury models, thereby exerting cardioprotective effects. Additionally, TLB ameliorated mitochondrial oxidative stress, protected the expression of copper ion transporter proteins, and modulated the expression of proteins involved in the lipoic acid pathway, thus conferring protective effects against cuproptosis.

The Traditional Chinese Medicine and Natural Products discussed above, together with their mechanisms of action and experimental models, are systematically summarized in Table 2.

## 5. Conclusions

Cuproptosis was once simplistically regarded as an incidental, toxicity-driven form of cell death triggered by excessive accumulation of copper ions. However, with the deepening of research, it has gradually been recognized that cuproptosis is actually the result of dysregulation in multiple pathways, including intracellular copper homeostasis imbalance, mitochondrial energy metabolism dysfunction, and aggregation of lipoylated proteins. The elucidation of the cuproptosis mechanism provides a new perspective for understanding neuronal death following stroke. By interfering with the mitochondrial respiratory chain and inducing proteotoxic stress, it plays a key role in ischemia–reperfusion injury, and the clinical value of related targeted therapies is gradually emerging.

Although researchers have made significant progress in their studies on cuproptosis, research on cuproptosis in IS is still in its early stages and many fundamental issues remain unresolved. In different brain cell types, does cuproptosis occur independently, or does it interact synergistically or antagonistically with other forms of cell death? What are the dynamic mechanisms of the copper homeostasis regulatory system during different phases of stroke? All these issues need to be clarified through meticulous experiments. Future studies should focus on employing single-cell sequencing and spatial transcriptomics technologies to precisely map the expression profiles of cuproptosis-related genes in stroke lesion areas, clarify their interaction networks with the immune microenvironment, promote clinical trials targeting key nodes of cuproptosis, and validate the synergistic efficacy of combining such approaches with traditional thrombolysis or neuroprotective agents, thereby laying the foundation for innovative therapeutic strategies in stroke.

## 6. Limitations and Perspectives

Emerging evidence suggests that copper dyshomeostasis and cuproptosis-related processes may contribute to ischemic brain injury, raising the possibility that cuproptosis could represent a potential therapeutic target for ischemic stroke (IS). However, the current evidence remains insufficient to fully establish a causal role for cuproptosis in ischemic brain injury. Most available studies are still preclinical, and the specific contribution of cuproptosis to neuronal injury is often difficult to distinguish from that of other forms of regulated cell death and concurrent pathological processes.

Recent animal studies have begun to provide functional evidence for the involvement of cuproptosis in cerebral ischemia/reperfusion injury. In particular, pharmacological induction of cuproptosis with elesclomol has been shown to aggravate neurological deficits and cerebral infarction in MCAO/R mice, supporting a potential contribution of cuproptosis to ischemic brain injury. However, the current evidence remains limited and is still largely based on pharmacological modulation and changes in cuproptosis-related markers. Further studies using genetic manipulation of key cuproptosis regulators, together with cell-type-specific and temporal analyses, are needed to establish a more definitive causal relationship.

In recent years, an increasing number of compounds have been reported to exert neuroprotective effects by modulating copper homeostasis or cuproptosis-related targets. Nevertheless, only a limited number of agents have been directly evaluated for their effects on cuproptosis in experimental models of cerebral ischemia. Although some candidate compounds have been shown to inhibit cuproptosis in other disease models, their therapeutic efficacy and underlying mechanisms in cerebral ischemia remain to be established. In addition, the precise molecular mechanisms and temporal dynamics of cuproptosis following cerebral ischemia remain incompletely understood. Most studies have focused primarily on acute injury, whereas the role of cuproptosis in long-term neurological recovery remains largely unclear.

Future studies should establish more rigorous and standardized criteria for identifying cuproptosis and combine pharmacological approaches with genetic manipulation to further clarify its causal contribution to ischemic brain injury. Greater attention should also be paid to the temporal dynamics and cell-type-specific mechanisms of cuproptosis following cerebral ischemia, as well as its interactions with other forms of regulated cell death. In addition, validation in clinical samples and assessment of long-term neurological outcomes will be necessary to determine whether targeting cuproptosis can ultimately be translated into an effective therapeutic strategy for IS. From a drug-development perspective, future studies should further evaluate promising cuproptosis-modulating agents in relevant cerebral ischemia/reperfusion models, with particular attention to their mechanisms of neuroprotection. Candidate compounds should be assessed not only for their ability to modulate cuproptosis, but also for their specificity, safety, and translational feasibility. Their ability to reach the brain at therapeutically relevant concentrations, including their blood–brain barrier permeability, as well as their potential compatibility with established reperfusion therapies, should also be carefully evaluated.

## Figures and Tables

**Table 1:** Small molecule drugs attenuating ischemic stroke injury by modulating Cuproptosis.

Drugs	Functional Mechanism	Experimental Model	Evidence for IS
Tetrathiomolybdate (TTM)	Chelates copper and reduces bioavailable Cu	rTg-DI CAA rats; isolated cerebral vessels	Indirect
Glutathione (GSH)	Chelates mitochondrial Cu^+^ and suppresses cuproptosis	PDAC cells; xenograft model	Indirect
Adrenomedullin (ADM)	Activates p38/MAPK–FOXO3 and suppresses FDX1-mediated cuproptosis	ccRCC cells; xenograft model	Indirect
Dexmedetomidine (DEX)	Suppresses FDX1-mediated cuproptosis and restores copper homeostasis	Rat MCAO/I-R; PC12 OGD/R	Direct
Edaravone dexborneol (EDB)	Downregulates SLC31A1/FDX1 and restores copper homeostasis	Mouse MCAO; neuronal OGD/R	Direct
8-Hydroxyquinolines (8HQs)	Promote copper transport and intracellular Cu accumulation	C6 glioma cells	Indirect
Zinc pyrithione (ZnPT)	Increases intracellular Cu, promotes DLAT aggregation and Fe-S protein loss	TNBC cells; xenograft model	Indirect

Note: Direct evidence refers to agents that have undergone comprehensive cuproptosis evaluation in IS models, or agents whose cuproptosis-related mechanisms have been validated in other disease models while showing therapeutic efficacy in cerebral ischemia. Indirect evidence refers to agents validated in non-stroke models but lacking sufficient evidence of therapeutic efficacy in cerebral ischemia.

**Table 2:** Natural products attenuating ischemic stroke injury by modulating Cuproptosis.

Drugs	Functional Mechanism	Experimental Model	Evidence for IS
Corymbinol A	Preserves Fe-S proteins and inhibits cuproptosis	Elesclomol/CuCl_2_-induced cuproptosis model	Indirect
Astragaloside IV (AS-IV)	Modulates the DUSP1–PHB2 axis and improves mitochondrial quality control	LPS-induced SCM mice; primary and HL-1 cardiomyocytes	Indirect
Calycosin-7-O-β-D-glucoside (CG)	Suppresses cuproptosis via the GSK-3β/ATF3/SLC31A1 pathway	SH-SY5Y OGD/R; rat MCAO/R	Direct
Phillygenin (PHI)	Promotes autophagy–lysosome-mediated CTR1 degradation and inhibits cuproptosis	Mouse MI/RI; H9c2 H/R; rat MCAO	Direct
Artesunate (ART)	Targets MT2A, regulates copper homeostasis, and inhibits cuproptosis	Cerebral I/R model; astrocytes	Direct
Xinfeng Capsule (XFC)	Regulates the METTL3/miR-221/222-3p/ATP7A axis and inhibits cuproptosis	Chondrocytes; rheumatoid arthritis model	Indirect
Trilobatin (TLB)	Targets FDX1 and inhibits cuproptosis	DOX-induced DIC mice; DOX-treated H9c2 cells	Indirect

Note: Direct evidence refers to agents that have undergone comprehensive cuproptosis evaluation in IS models, or agents whose cuproptosis-related mechanisms have been validated in other disease models while showing therapeutic efficacy in cerebral ischemia. Indirect evidence refers to agents validated in non-stroke models but lacking sufficient evidence of therapeutic efficacy in cerebral ischemia.

## Data Availability

Data are contained within the article.

## Abbreviations

The following abbreviations are used in this manuscript:

IS	Ischemic stroke
TCA	Tricarboxylic acid
Fe-S cluster	Iron–sulfur clusters
DALYs	Disability-adjusted life years
ATP	Adenosine triphosphate
ROS	Reactive oxygen species
BBB	Blood–brain barrier
ER	Endoplasmic reticulum
CTR1	Copper transporter 1
CCS	Copper chaperone for superoxide dismutase
SOD1	Superoxide dismutase 1
ATOX1	Antioxidant 1 copper chaperone
ATPases	Adenosine triphosphatases
ATP7A	ATPase copper transporting alpha
ATP7B	ATPase copper transporting beta
TGN	Trans-Golgi network
MT	Metallothionein
GSH	Glutathione
COX17	Cytochrome c oxidase assembly protein 17
COX	Cytochrome c oxidase
SLC31A1	Solute carrier family 31 member 1
FDX1	Ferredoxin 1
LIAS	Lipoyl synthase
HSP70	Heat shock protein 70
DLAT	Dihydrolipoamide S-acetyltransferase
DLST	Dihydrolipoamide S-succinyltransferase
RAB18	Ras-related protein Rab-18
CPT1A	Carnitine palmitoyltransferase 1A
DLD	Dihydrolipoamide dehydrogenase
PPP1R15A	Protein phosphatase 1 regulatory subunit 15A
PP1	Protein phosphatase 1
4EBP1	Eukaryotic translation initiation factor 4E-binding protein 1
G6PD	Glucose-6-phosphate dehydrogenase
CREB	cAMP response element-binding protein
BDNF	Brain-derived neurotrophic factor
I/R	Ischemia/reperfusion
MCAO/R	Middle cerebral artery occlusion/reperfusion
METTL14	Methyltransferase-like 14
TTM	Tetrathiomolybdate
CAA	Cerebral amyloid angiopathy
Aβ	Amyloid-β
Dex	Dexmedetomidine
EDB	Edaravone dextrose
MCAO	Middle cerebral artery occlusion
OGD/R	Oxygen–glucose deprivation/reperfusion
SLC7A11	Solute carrier family 7 member 11
8HQs	8-Hydroxyquinolines
ZnPT	Zinc pyrithione
TCM	Traditional Chinese medicine
ACO2	Aconitase 2
AS	Astragaloside IV
DUSP1	Dual specificity phosphatase 1
PHB2	Prohibitin 2
MQC	Mitochondrial quality control
CG	Calycosin-7-O-β-D-glucoside
AR	*Astragali Radix*
GSK-3β	Glycogen synthase kinase 3β
ATF3	Activating transcription factor 3
PHI	Phillygenin
ART	Artesunate
MyD88	Myeloid differentiation primary response 88
NF-κB	Nuclear factor-kappa B
MT2A	Metallothionein 2A
XFC	Xinfeng Capsule
METTL3	Methyltransferase-like 3
miR-221/222-3p	MicroRNA-221/222-3p
TLB	Trilobatin
DIC	Doxorubicin-induced cardiotoxicity
